# A pilot survey of post-deployment health care needs in small community-based primary care clinics

**DOI:** 10.1186/1471-2296-12-79

**Published:** 2011-07-29

**Authors:** Polly H Noël, John E Zeber, Mary J Pugh, Erin P Finley, Michael L Parchman

**Affiliations:** 1 VERDICT, Central Texas Veterans Health Care System Scott & White, 7400 Merton Minter Blvd, San Antonio, Texas 78229 USA; 2 University of Texas Health Science Center at San Antonio, 7703 Floyd Curl Drive, San Antonio, Texas 78229 USA; 3 Agency for Healthcare Research & Quality, 540 Gaither Road, Rockville, MD 20850 USA

**Keywords:** Primary care, Post-deployment health, PTSD, Depression

## Abstract

**Background:**

Relatively little is known regarding to what extent community-based primary care physicians are encountering post-deployment health care needs among veterans of the Afghanistan or Iraq conflicts and their family members.

**Methods:**

This pilot study conducted a cross-sectional survey of 37 primary care physicians working at small urban and suburban clinics belonging to a practice-based research network in the south central region of Texas.

**Results:**

Approximately 80% of the responding physicians reported caring for patients who have been deployed to the Afghanistan or Iraq war zones, or had a family member deployed. Although these physicians noted a variety of conditions related to physical trauma, mental illnesses and psychosocial disruptions such as marital, family, financial, and legal problems appeared to be even more prevalent among their previously deployed patients and were also noted among family members of deployed veterans.

**Conclusions:**

Community-based primary care physicians should be aware of common post-deployment health conditions and the resources that are available to meet these needs.

## Background

Since the initiation of Operations Enduring Freedom and Iraqi Freedom (OEF/OIF), more than 2 million active duty military, military reserve, and national guard members have deployed to Afghanistan or Iraq [1]. Of these, over 40,000 have been killed or wounded in action [2]. Although improvements in battlefield armor, casualty evacuation, and medical care have enhanced survival rates following combat injuries, many survivors of once-lethal injuries now require prolonged medical and rehabilitation care [3-5]. In addition, as many as 1 in 5 veterans who served in Iraq and Afghanistan report symptoms of post-traumatic stress disorder (PTSD), depression, and other mental health problems [6-11]. Forty percent of those who have served in Afghanistan or Iraq have deployed more than once, increasing the risk of developing deployment-related mental health conditions, which rises with the frequency of re-deployments [1,12,13]. Furthermore, individuals deployed to Afghanistan and Iraq are on average older than those deployed in previous wars, and therefore also more likely to develop musculoskeletal injuries and chronic illnesses in theatre, such as cardiovascular disease, diabetes, and chronic pain [6,14-18].

The Veterans Healthcare Administration (VHA) was created to provide medical care for veterans who were injured or developed an illness during their military service [19]. Services have expanded to cover economically disadvantaged veterans, while other veterans can receive basic health care services for a co-payment. Although more recent policy changes provide up to 5 years of free health care to OEF/OIF veterans who enroll within 5 years of separation from military service, only about 35% of those who have separated from military service have sought health care in the VHA [6,20]. The combination of significant morbidity and low transition rates to the VHA suggests that veterans not seeking care within the VHA may instead be turning to community-based providers for their medical and/or mental health needs.

In addition, more than 120,000 civilian contractors who have deployed in Afghanistan or Iraq are not eligible for benefits from either the Department of Defense (DoD) or VHA, even though more than 4,000 of them have been injured or killed while deployed [21]. The spouses, children, and other dependents of veterans of the Afghanistan and Iraq wars have also been found to be at increased risk of depression, substance abuse and other mental health problems during and after deployments [22-28]. As these family members are largely ineligible for care within the VA system, they are also likely to seek care in the community. The objective of this pilot study was to explore the experiences of community-based primary care physicians in treating veterans and their family members with post-deployment health care needs.

## Methods

### Study Design, Setting, and Participants

We conducted a cross-sectional survey of primary care physicians working at clinics belonging to the South Texas Ambulatory Research Network (STARNet), a practice-based research network [29]. These unaffiliated small urban and suburban practices, each with 1 or 2 family physicians or general internists, serve a population of primary care patients diverse in demographic characteristics, insurance coverage, and health care needs. We excluded any physicians who provided contract services for the VHA. Sixty physicians active in STARNet during the time of the study met inclusion criteria for participation.

### Data Collection

We created a brief, 9-item survey to assess whether any of the physicians' patients ever reported serving in Afghanistan or Iraq since 2001 in any of the following capacities: active duty military, military reserve, National Guard, civilian contractor, journalist, or other role. Two items asked the physicians to specify the number of patients seen in the last 12 months who reported: 1) being deployed to Afghanistan or Iraq, or 2) having a spouse/partner or close family member who had been deployed. One item assessed 20 categories of problems frequently experienced by individual who have been deployed. These included combat- and non-combat injuries, infectious diseases, sexual trauma, psychiatric disorders, and psychosocial disruptions such as marital, family, and legal or financial problems. Another item assessed 5 categories of psychiatric and psychosocial problems that may have been experienced by family members of those who had been deployed: depression, alcohol or substance abuse, marital difficulties, family problems, and legal or financial problems). Additional items assessed the number of years that physicians had been in practice at the STARNet clinic, the total number of unique patients they treated each year at the clinic, whether they accepted TRICARE insurance (i.e., DoD's health insurance plan for active duty members, National Guard and Reserve members, retirees, and dependents that includes purchased care from civilian providers), and their interest in obtaining continuing education on post-deployment health-related conditions. The survey was distributed by the STARNet coordinator and a research associate to the physicians in person, by mail, fax, and/or e-mail in accordance with their preferences.

The research reported here was conducted in compliance with the Helsinki Declaration. It was reviewed and approved (#HSC20090379E) by an independent ethics committee, the Institutional Review Board (IRB) of the UT Health Science Center at San Antonio. An IRB-approved information sheet informing participants of their rights as research subjects was attached to the questionnaire.

### Analytic Plan

The surveys were formatted with Teleform^® ^software. Completed surveys were reviewed by trained analysts and scanned into an electronic database. Analyses consisted of univariate statistics to describe the sample and document the proportion of physicians who had seen or treated patients with post-deployment health care needs. The prevalence and diversity of physical and mental health problems noted by the physicians among their patients was also summarized.

## Results

Thirty-seven of 60 physicians returned surveys, yielding a response rate of 61.6%. Respondents indicated that they had been in practice at the STARNet clinics an average (SD) of 12.2 (9) years. Approximately 52% reported caring for more than 1,000 unique patients yearly while 48% cared for 1,000 or fewer patients. Thirty (81%) of the 37 responding physicians reported having 1 or more patients who had informed them that they had been to Afghanistan or Iraq since 2001. Of these, 19 (63%) had seen patients who had been deployed as active duty military, 10 (33%) had seen Military Reservists, 9 (30%) had seen members of the National Guard, 15 (50%) had seen civilian contractors, and 3 (10%) had seen journalists. The majority of the responding physicians (N = 22, 59%) had seen 10 or fewer patients in the prior 12 months who had informed them that they had been deployed to Afghanistan or Iraq, while 5 additional physicians (14%) reported seeing more than 10 of these patients in the last year alone.

In general, the physicians were more likely to report psychiatric or psychosocial problems, as opposed to physical conditions, among their patients who had been deployed (Table 1). Fourteen physicians reported seeing patients with post-deployment related depression, PTSD, alcohol or substance abuse, or marital or family problems. In contrast, 9 physicians reported caring for patients with one or more of the following deployment-related medical conditions: non-combat injury, traumatic brain injury (TBI) or other neurological problem, wound care or re-infection, orthopedic problem related to traumatic injury, hearing loss/tinnitus, or medical illness that developed or worsened during deployment. None of the physicians reported seeing patients with deployment-related amputations or prosthetic devices, sexual trauma, acinetobacter or leishmaniasis, vision loss, or depleted uranium exposure.

**Table 1:** Post-deployment medical and psychological problems noted among individuals deployed to Afghanistan or Iraq seen by community-based primary care providers (N = 30).

Deployment-related Problem	N (%)
Traumatic amputation and/or prosthetic device	0

Wound care or re-infection	3 (10%)

Other orthopedic problem related to combat or burn injury	2 (7%)

Traumatic brain injury	2 (7%)

Other neurological problem related to head injury (e.g., epilepsy)	1 (3%)

Non-combat injury that occurred during deployment (e.g., motor vehicle accident)	3 (10%)

Vision loss	0

Hearing loss, tinnitus	2 (7%)

Medical illness that developed or worsened during deployment	4 (13%)

Exposure to depleted uranium	0

Acinetobacter or leishmaniasis	0

Other infectious disease	1 (3%)

Military sexual trauma	0

Post-traumatic stress disorder	6 (20%)

Depression	9 (30%)

Alcohol or substance abuse	4 (13%)

Marital problems	6 (20%)

Family problems	2 (7%)

Legal or financial problems	0

Twenty-nine (78%) of the responding physicians also reported encountering 1 or more patients in the prior 12 months who had told them that their spouse or partner or other close family member had been deployed to Afghanistan or Iraq. Seventeen (58%) of these physicians reported treating or referring these family members for depression, alcohol or substance abuse, and/or marital, family, or legal/financial problems. Fifty-one percent (N = 19) of the responding physicians indicated that they were interested in receiving continuing medical education on one or more topics related to post-deployment health. Fifteen expressed interest in learning more about general post-deployment health, 14 expressed interest in PTSD, and 8 indicated they wanted to learn more about TBI.

## Discussion

The results of this study indicate that the majority of community-based primary care physicians who responded to our survey are providing care to patients who have either been deployed to the Afghanistan or Iraq war zones or had a family member deploy. Although these physicians noted a variety of conditions related to physical trauma, mental illnesses and psychosocial disruptions appeared to be more prevalent among their previously deployed patients and were also noted among family members of deployed veterans. In spite of efforts to encourage seamless transition from the DoD health care system to the VA, which include providing 5 years of free care for all veterans of the Afghanistan and Iraq conflict, [6] these results suggest that some veterans eligible for care in the VA are instead seeking care in community-based primary care clinics. Although many veterans may have access to private health insurance or simply prefer non-VA care, community-based physicians who care for them should be aware of the special needs of veterans, especially those who served in combat zones.

Community-based physicians may feel confident in their ability to coordinate or treat the physical sequelae of deployment-related trauma, but appear to face greater uncertainty when attempting to manage the mental health and psychosocial consequences of deployment. This may be especially true regarding PTSD, which was the most frequently selected continuing medical education topic the providers expressed interest in after post-deployment health issues in general. Referral options for deployment-related conditions such as PTSD may be limited because of the relative lack of mental health providers in the community trained to provide evidence-based treatments, especially in rural settings, and because some private insurance plans do not offer parity in mental health coverage [6]. In addition, studies indicate that OEF/OIF active duty personnel and veterans may avoid seeking treatment for conditions such as PTSD or depression because of stigma and negative perceptions about mental health care [6,30,31].

It may therefore be helpful for community-based physicians who encounter patients with post-deployment health care needs to be aware of the specialized services and resources available though the VHA and other agencies dedicated to caring for veterans and their families. In spite of initial problems that arose during the early years of the Afghanistan and Iraq conflicts when the VA was overwhelmed by the large number of traumatized veterans seeking care, the VA has dramatically increased resources and services for OEF/OIF veterans and made a concerted effort to provide state-of-art care for conditions such as traumatic brain injury and PTSD. Community-based primary care physicians therefore should consider the system of coordinated care available to all former military, reservists, and National Guard members who served in Afghanistan or Iraq through the VHA, especially for those who have mental health conditions and limited mental health benefits. In addition, several short screening measures that have been validated for use among military personnel recently returned from combat and other provider resources are available to the public at the VA's National Center for PTSD website http://www.ptsd.va.gov/professional/index.asp.

The Department of Veterans Affairs also operates a system of Vet Centers that are organizationally separate from VA medical centers [32]. The Vet Centers provide readjustment counseling and outreach services to combat veterans and their families. In addition, a recent report prepared by the RAND Corporation has catalogued a number of non-federal resources available to OEF/OIF veterans and their families [6]. These include a number of nationwide and regional programs such as the Coming Home Project and Operation Comfort that provide support and free counseling or psychotherapy for returning OEF/OIF veterans and their families, state-sponsored programs to assist veterans with their mental health needs, and university counseling services designed specifically for veteran students [6].

Fewer programs and resources are available, however, to government contractors who served in Afghanistan and Iraq. In spite of having similar experiences and conditions as military combat personnel, they are not authorized to receive care from Department of Defense or VA medical facilities. With the exception of contractors who are also veterans or retired military, community-based physicians and mental health providers are likely the only options that government contractors have. Although the Defense Base Act requires that defense contractors provide workers' compensation insurance for civilian employees who work in war zones, recent news reports indicate that about half of the claims for PTSD that have been filed by government contractors have been denied or challenged by insurers [33,34]. Virtual support groups for government contractors have started to appear on the world-wide web, but it is imperative we begin to develop additional resources for these civilian veterans.

There are several limitations in the interpretation of findings from this study, including the relatively small sample size. Because the metropolitan area in which the clinics were located is home to several military bases, as well as National Guard and Reserve units, and is also noted for a large population of retired veterans, these results may not generalize to other regions of the country that have less of a military presence. We did not attempt to corroborate providers' self-reports with chart reviews and it is possible that their retrospective recall of post-deployment exposure or deployment-related health concerns may have been subject to memory bias. This study, however, has provided a preliminary glimpse into post-deployment health care needs encountered by physicians in community-based primary care clinics.

## Conclusions

Although the problem of detecting and providing appropriate assistance for post-deployment mental health problems among military personnel and their spouses among military primary care settings has been previously noted, [22,35] this research focused on post-deployment health care needs encountered by primary care physicians in non-military, community-based primary care clinics. The post-deployment health priorities identified by physicians in this study are consistent with those identified in our prior work among patients seeking care in the same non-VA clinical network - namely, the central importance of addressing psychological and psychosocial concerns post-deployment, and the cumulative effect of deployment and post-deployment stressors on the health of family members as well as individual veterans [36]. More research is needed comparing outcomes of those who served in Iraq or Afghanistan and who are cared for in the VA to those who receive care in non-VA community settings. Additional research is also needed on the ability of these different groups of veterans to get the care they need, and the social and emotional impact of their deployment on their families and communities. Primary care physicians remain the first and only point of contact for comprehensive health care for many of those who have served their country. These data suggest that community-based providers should be aware of common post-deployment health conditions and the resources that are available to meet these needs.

## Competing interests

The authors declare that they have no competing interests.

## Authors' contributions

PHN, MLP, MJP, and JEZ conceptualized the study. PHN, JEZ, and MLP obtained funding for the study. JEZ supervised data collection. PHN and JEZ directed data analysis. PHN drafted and finalized the manuscript. All authors contributed to interpreting study results and writing the manuscript. All authors read and approved the final manuscript.

## Pre-publication history

The pre-publication history for this paper can be accessed here:

http://www.biomedcentral.com/1471-2296/12/79/prepub
